# Report of a Case of Placenta Prævia Centrales with Transverse Position of the Fœtus, and Quick Recovery

**Published:** 1890-12

**Authors:** W. W. Stewart

**Affiliations:** 109 East 12th Street; New York


### REPORT OF A CASE OF PLACENTA PRÆVIA CENTRALES WITH TRANSVERSE POSITION OF THE FŒTUS, AND QUICK RECOVERY.

BY W. W. STEWART, M. D., NEW YORK.

This case strikes me as interesting, not only from the abnormal position of the fœtus and placenta, but on account of the quick convalescence from the confinement.

Mrs. K., age 38, has had six living children; all confinements prior to the last one without any complications and comparatively easy.

She first came under my care on April the 14th, 1890, when she came to me for treatment of varicose veins of right leg, for which she received treatment and was much benefited.

At this time she spoke to me of her expected confinement, and engaged me to attend her. She expected to be confined the first of September. On August the 20th I was called at 3 o'clock in the morning, and on arriving found that she had no pains, but a severe hemorrhage, which had soaked through the bedding and formed quite a pool on the floor.

On examination, the cervix had a peculiar velvety feel. The os externum was quite rigid, and with difficulty would admit the point of the index finger. The os internum was impervious. Ballottement was indistinct, and a marked pulsation could be felt with the point of the finger.

She complained of feeling life to an unusual degree, and on palpation the fœtus could be felt every few minutes to make a complete revolution with one spring, then rest for a minute and commence its struggles again, as one who was being suffocated.

The vagina was well plugged with borated cotton pledgets, fastened together with a piece of twine, and well soaked with glycerine, and one-fourth grain of morphia, given hypodermically. She slept well until ten in the morning, when she awoke complaining of intense thirst and weakness. Milk punches were ordered every two hours, and milk *ad libitum.* Pulse was then 110, temperature 101 degrees

On careful examination, the diagnosis of placenta prævia centrales, which had been made, was confirmed, and she was advised to have labor brought on at once, which she refused to have done. The vagina was then well douched and again plugged, no hemorrhage having occurred in the meanwhile.

This treatment was continued for four days, after which the tampons were not replaced.

She was then furnished with tampons of borated cotton, and instructed if any hemorrhage should occur, to tampon her vagina well, and send for me.

From this time until September the 15th weekly visits were made, and patient was kept on tonics and good food.

On September the 15th I was called again, and found her complaining of incapacity to hold her water, and a heavy feeling in the lumbar region, but no pains.

On examination, the os was found dilated sufficiently to allow the finger to pass the os internum, and the lobulated placenta could be well defined.

The fœtus occupied the transverse position, back to the front, head on a level with the crest of the ileum of right side.

Liquor amnii excessive.

September the 16th I called again, and made another examination with Dr. Proben, who confirmed my diagnosis and asassisted me in the subsequent delivery.

After the examination, the hemorrhage began the second time and it was decided to bring on labor at once. She was placed on a table and chloroform administered.

The vagina was then douched with 1 to 6000 bichloride; the hand, well oiled, was then passed into the vagina and cervix quickly dilated with the fingers. At this time, and during the detachment of the placenta, the hemorrhage was alarming. When the os was sufficiently dilated, the hand was passed into the uterus and the placenta dissected off on all sides, the membranes ruptured and podalic version performed.

The uterus being well followed down, the child was extracted. The legs, body and shoulders were delivered without difficulty. When the neck came into view, the funis was found tightly wound around the child's neck, and the chin became fastened under the arch of the pubes. Forceps were applied to the aftercoming head and delivered.

When born, the child was blue, and made no respiratory efforts. Ice and hot water, alternately with mouth to mouth insufflation, artificial respiration and smart slaps soon provoked respiratory efforts. The child was then wrapped in warm flannel and laid to one side.

In the meantime Squibb's ergot ʒii had been given to the mother, and the uterus well kneaded; hot bichoride, 1 to 6000, was injected into the uterus, and pieces of ice slipped into its cavity, which soon controlled the hemorrhage. On examination of the membranes, they were found not intact, and the hand was again passed into the uterus and a strip of membrane six inches in length was found in the left cornua of the uterus and removed. The uterus was again douched with 1 to 6000 bichloride, and parts well washed and binder applied with vulva pad of bichloride gauze 1-6000, which was to be changed every three hours. One drachm of ergot was administered in thirty minutes, as the uterus was not sufficiently contracted, and morphia, grain one-fourth, with whisky, was administered hypodermically.

Pulse quite weak and rapid; milk punch given by the mouth, which, with the morphia, soon brought the pulse up in strength and produced a fall from 140 to 110, temperature 101 degrees. Vagina was douched with bichloride, 1 to 6000, for ten days, three times a day. Urine drawn twice daily and once at night. Bowels moved on the fourth day. Whisky and milk ordered every two hours. Patient discharged on the tenth day; was put on tonic treatment, to be continued as long as necessary.

The following is the chart of temperature for period of convalescence:

September 16—12 p. m., temperature, 101 deg.; 1:40 p. m., pulse, 110.

September 17—9 a. m., temperature, 101 1-10; pulse, 114; 6 p. m., temperature, 101; pulse, 110.

Septemper 18—9 a. m., temperature, 101 1-5; pulse, 108; 7 p. m., temperature, 100; pulse, 108.

September 19—8 a. m., temperature, 100; pulse, 100; 5 p. m., temperature, 100 1-10; pulse, 109.

September 20—9 a. m., temperature, 101; pulse, 105; 6 p. m., temperature, 100; pulse, 102.

September 21—8 a. m., temperature, 99; pulse, 90; 5 p. m., temperature, 100; pulse, 98.

September 22—8 a. m., temperature, 100; pulse, 99; 7 p. m., temperature, 98; pulse, 90.

September 23—9 a. m., temperature, 98; pulse, 90; 8 p. m., temperature 98 1-2; pulse, 92.

September 24—8 a. m., temperature, 99; pulse, 92; 6 p. m., temperature, 100; pulse 95.

September 25—8 a. m., temperature 98; pulse, 90; 7 p. m., temperature, 98; pulse, 92. Patient discharged.

*109 East 12th Street.*

